# Culture and epidemiology of diabetes in South Asia

**DOI:** 10.7189/jogh.09.020301

**Published:** 2019-12

**Authors:** Bipin Adhikari, Shiva Raj Mishra

**Affiliations:** 1 Nepal Community Health and Development Centre, Balaju, Kathamndu, Nepal; 2 Centre for Tropical Medicine and Global Health, Nuffield Department of Medicine, University of Oxford, Oxford, UK; 3 Nepal Development Society, Chitwan, Nepal

Disease epidemiology has a deeper relationship with the dynamic nature of culture [1]. Health behaviors in general are largely shaped by the cultural norms and customs in a society. A mere identification of a behavior could be only a layer on the outer sphere of a particular disease epidemiology and the interventional efforts to counteract such behaviors through for example public health measures could be futile and volatile, unless the deeper cultural factors are addressed [2].

Anthropological methods, such as ethnographical studies are useful in disentangling such cultural factors underlying the disease [1]. Cultural factors are, however, unique to a particular community and it requires a combination of formative research on the local social, cultural and health system factors, and collaboration with stakeholders for the design of an intervention [3,4].

The Diabetes epidemic in South Asia is projected to reach 134 million by 2050 [5], halting this rapid growth in burden is impossible without understanding the cultural underpinnings behind the particular health, food and lifestyle related behaviors. Tailoring the interventions based on cultural epidemiology can be promising. Understanding culture needs greater engagement with the community for an effective translation of epidemiological findings which can ultimately help to design a culture specific intervention, in addition to the advocacy and awareness [4]. Existing network of community health workers can become the conduit of local culture for the appropriateness of interventions [4,6]. As has been shown by a recent community based trial that utilized the existing community health workers in monitoring and managing hypertension [6], strategies inherent in community engagement [4] wherein existing health workers including traditional healers can be trained and devolved to early diagnosis, monitor and promote the healthy life style is promising initial steps to fill the gaps in cultural epidemiology of Diabetes and other life style based diseases.

While there is already an amplitude of information on the drivers of Diabetes in South Asian region, much less is known about its cultural-epidemiology – a nascent discipline that describes both the nature and the distribution of illness experiences, meanings, and behaviours [7]. Despite growing burden recent evidence highlighted inadequate strategies being placed for management, or management not being effective [5]. However, broader context of Diabetes epidemic where the influence of culture in food and lifestyle unique to South Asia remained relatively overlooked. Here in this piece, we draw reader’s attention to the cultural-epidemiology of Diabetes and explain its implication on its control and management in South Asia.

## SOUTH ASIA AND DIABETES

The current trend of gestational Diabetes, female’s higher odds of developing Diabetes and being obese clearly prompts to explore the deeper underpinnings within the South Asian culture. A recent COBIN-D baseline study explored the burden of Diabetes in a local community of Western Nepal to be nearly 12% and abdominal obesity to be 80% [8]. Factors associated with higher burden, overall low control and adherence to medication was older age (≥35 years), ethnic minorities, abdominal obesity, being overweight or and being obese and hypertensive [8]. These epidemiological factors require an exploration of cultural underpinnings.

In South Asia, females are mostly confined to indoor household works, and are largely restricted to participate in social activities including outdoor physical activities [9]. Participating in outdoor activities for females without a male company is considered a security threat particularly due to potential sexual abuse such as harassment and rapes in public. In addition, even if they are encouraged to do so, females are hesitant to participate in physical activities, particularly due to stigma attached to females’ outdoor activities seen as “outgoing” with negative social connotations. Similarly, the lack of outdoor spaces (for example public parks) for physical activities, further restricts both male and female gender. Bearing these cultural elements in mind, simple encouragement prescribed by health workers and policy makers do not seem to bring the changes.

Looking at broader life style factors, sugar and sweets occupy a special place in Hindu religion and are offered to gods and goddesses. Historical discovery of sugar in India and its spread to the west further adds to the current understanding of Indian food culture where sugar and sweets are considered as the symbol of warmth and are a welcome gesture to a guest. An Indian tea, a frontline gesture to welcome guests, is invariably served with sugar and milk. A lack of addition of sugar and milk in the tea is considered against the tradition and may even connote either the low socio-economic status or inadequate respect to the guests. In South Asian culture, rejecting such offerings (for example: sweets and sweet tea) as a guest is considered as disrespectful and thus may perpetuate the cycle of (non-)reciprocity. These considerations are important for designing interventions to support lifestyle modification and glucose monitoring.

South Asia is also at the central stage of increasing urbanization, economic prosperity and rural to urban migration. With increasing economic prosperity, population in South Asia are vulnerable to reduced energy expenditure and increased sedentary behavior. The food in South Asia is rich in high refined carbohydrates, sweets and saturated fats. In addition, the recent trend of rural to urban migration and rising economic prosperity have increased the affordability conducive to high consumption of such foods, affecting all population and in particular the indigenous ethnic groups. In indigenous/tribal population, the improved economy and rural to urban migration has replaced their staple food (low in fat and high in fibre) such as millet, barley, buckwheat, tubers to rice along with the transition from ancient labor intensive subsistence farming to rice based farming [5,10]. Abandoning subsistence farming where the energy expenditure is high coupled with adopting the high energy diet in urban areas who work in low wage employment system can certainly explain their vulnerability to Diabetes.

Another important aspect of culture is the stereotypes that shape the stigma around the disease. Diabetes is stigmatized as a lifelong condition, considered as unhealthy and affects both gender invariably on marriages. The extent of stigma may be compounded when it comes to finding a husband for a women, particularly because of high patriarchy in South Asia. A person with diabetes together with obesity may further incur discrimination in finding a job. Similarly, taking medicine is perceived as a sign of “not healthy” and “weak”. Stigma around injectable drugs such as insulin are even higher and may play a critical role in discerning the uptake and adherence of anti-diabetic medicines. Reducing stigma through engagement of patients, relatives and both formal and informal health care providers can promote the early health seeking behavior [11].

## CULTURALLY TAILORED INTERVENTIONS

Mitigating the burden of diseases including diabetes require a tailoring of the interventions by the health system based on the existing cultural practices. Similar to many other developing low and middle income countries where traditional medicine serves the community in the fore front, in South Asia, ancient Ayurvedic medicine and other folk healing practices are attended by huge proportion of population. These practices, although, adequately not proven to have effect in Diabetes, continue to attract people with chronic illnesses including Diabetes and may exacerbate the disease progression by delaying the health seeking behaviour.

**Figure:**
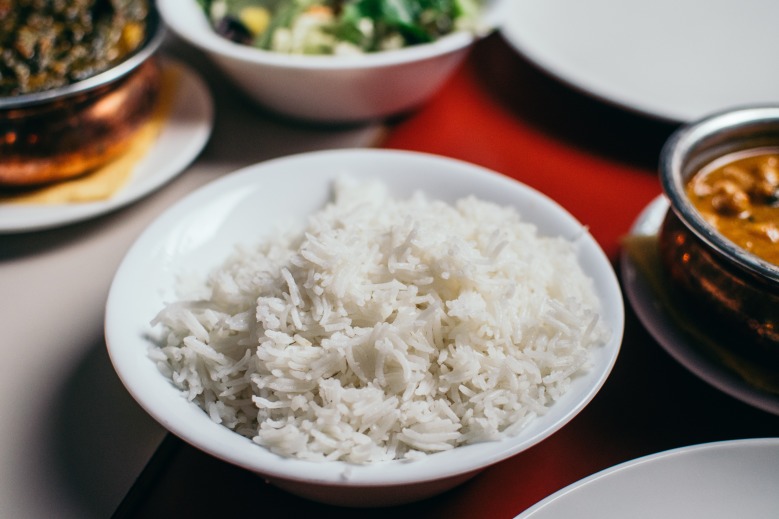
Photo: Rice is the main dish in South Asia. The image is downloaded from unsplash.com, originally contributed by Pille-Riin Priske, @pillepriske.

The traditional healers can be trained to identify the early symptoms and signs such as classical “3Ps” (Polyuria, Polydipsia, and Polyphagia) in patients so that they can facilitate their early diagnosis and treatment at the health facilities. In addition, benefits of Yoga which has shown to have impact on glycemic control in Diabetes when compared to regular exercise, therefore, integrating it will simply improve the healthy life style.

## WAY FORWARD

Disease epidemiology feeds into the information system for health policy and interventions. In recent years, although there are increasing efforts directed towards understanding the cultural underpinnings for the design of interventions, much less has been in application. Diabetes is inextricably linked with the lifestyle factors which are mostly rooted to the cultural practices. Interventions directed to discourse the current rising trend of Diabetes in South Asia require a cultural tailoring. Future interventions including the responses from health systems can benefit by 1) exploring the factors embedded in local social and cultural context through formative research, 2) tailoring the interventions based on the findings of formative research, and 3) ultimately engaging with both formal and informal health care providers for the implementation. Future research should focus on operational aspects of exploring such an approach including their impacts on Diabetes epidemiology, and relevance to the design of interventions, policy and responses by health systems.
